# Understanding Youth Online Experiences and Mental Health: Development and Validation of the Digital Activity and Feelings Inventory (DAFI)

**DOI:** 10.1002/mpr.70028

**Published:** 2025-06-17

**Authors:** Katarzyna Kostyrka‐Allchorne, Jake Bourgaize, Aja Murray, Mariya Stoilova, Iqra Abbas, Amy Bridgwood, Eliz Azeri, Chris Hollis, Ellen Townsend, Sonia Livingstone, Edmund J. S. Sonuga‐Barke, the Digital Youth Research Programme

**Affiliations:** ^1^ Department of Psychology School of Biological and Behavioural Sciences Queen Mary University of London London UK; ^2^ Department of Child and Adolescent Psychiatry Institute of Psychiatry Psychology & Neuroscience King's College London London UK; ^3^ Clinical Outcome Solutions Folkestone UK; ^4^ School of Psychology University of Edinburgh Edinburgh UK; ^5^ Department of Media and Communications London School of Economics and Political Science London UK; ^6^ Department of Psychology Institute of Psychiatry Psychology & Neuroscience King's College London London UK; ^7^ National Institute of Health Research (NIHR) MindTech MedTech Research Centre Institute of Mental Health School of Medicine University of Nottingham Nottingham UK; ^8^ School of Psychology University of Nottingham Nottingham UK

**Keywords:** adolescent, digital activity, mental health, screen time, wellbeing

## Abstract

**Objectives:**

We created the Digital Activity and Feelings Inventory (DAFI) to measure youth digital activities and the psychological reactions they evoke, established its psychometric properties and tested its validity in predicting mental health relative to screen time estimates.

**Methods:**

An initial pool of items was generated using the existing research on youth digital activity and mental health and further refined via consultations with experts and young people (online youth panel sessions, *n* = 14). The participants (*n* = 383, mean age = 19 years) completed the resulting DAFI alongside established measures of depression, anxiety, wellbeing, and screen time. The DAFI factor structure, reliability and predictive validity were tested.

**Results:**

Exploratory factor analyses identified five digital activity subscales: *Risky Content*, *Risky Interactions*, *Social Comparison, Leisure Activities and Social Engagement* and three psychological reactions subscales: *Negative Self‐Reactions, Negative Stress Reactions*, and *Positive Reactions*. Internal consistency and test‐retest reliability were high. *Social Comparison* and *Negative Self‐Reactions*, but not screen time, independently predicted depression and anxiety symptoms. *Positive Reactions*, lack of *Negative Self‐Reactions*, lower screen time and *Social Engagement* predicted wellbeing.

**Conclusion:**

The DAFI is a reliable measure of digital activities and associated psychological reactions and predicts youth mental health better than screen time.

## 1 Introduction

It has been proposed that the increased use of digital technology is contributing to the decline in adolescents' mental health observed over recent decades across Europe and North America (Twenge et al. 2020). Studies have reported statistically significant, though typically weak, associations between digital activity and poor mental health (National Academies of Sciences et al. 2024; Santos et al. 2023). However, the meaning of these links remains difficult to determine (Dickson et al. 2018; Kucirkova et al. 2023; Orben et al. 2024) mostly due to the limitations of the existing measures (Browne et al. 2021) and the lack of theoretical models precluding the granular analysis necessary to inform policy and practice (Christakis 2019; Livingstone 2021). First, most studies characterise digital activity using the unidimensional construct of screen time (i.e., time spent using screen‐based devices in a typical day/week), which fails to distinguish between different types of digital activity and the degree of risk or protection they confer (Jaycox et al. 2024). Second, it does not consider the subjective reactions evoked by the digital activity, which is a substantial gap given the literature demonstrating that the subjective experience of an event within a specific context influences its mental health impact (Herres et al. 2016; Wichers et al. 2009). Based on these insights, we have recently hypothesised that engaging in specific risky digital activities increases the risk of depression by evoking frequent and persistent negative affective (e.g., anger) and cognitive reactions (e.g., ‘I feel stupid’). These effects are then exacerbated when depression levels further increase both risky digital activity and the negative affective and cognitive reactions to it (Sonuga‐Barke et al. 2024).

To address the measurement limitations of the previous literature, and test the above hypothesis, we developed the *Digital Activities and Feelings Inventory* (DAFI), a self‐report questionnaire co‐designed with a youth panel of adolescents aged 12–17 years. The DAFI was designed specifically to distinguish digital activities that may be risky to mental health and psychological wellbeing from those that are either neutral or protective in this context. Using the DAFI, study participants report on their digital activities in the past 2 weeks: (i) their specific digital activities and (ii) the affective and cognitive reactions (hereafter ‘psychological reactions’) being online has induced. Here, we describe the development and psychometric properties of the DAFI and compare it with a generic measure of screen time on how well each predicts mental health. Specifically, we (i) report the frequency of specific digital activities and psychological reactions that encompass both affective and cognitive dimensions (Sonuga‐Barke et al. 2024); (ii) explore the latent structures and the reliability with which these constructs can be measured; and (iii) use multiple regression models to identify which aspects of digital activity (including screen time) and psychological reactions best predict mental health.

We hypothesised that risky digital activity increased symptoms of depression and anxiety and reduced wellbeing by evoking more frequent negative psychological reactions to being online. We also hypothesised that the DAFI would provide greater predictive power in detecting variations in all outcome variables compared with a screen time measure.

## 2 Methods

### 2.1 Initial Development and Refinement of the DAFI Prototype

To ensure the DAFI reflected young people's online experiences and used relevant and comprehensible language, we worked with a youth panel during its prototype development. Youth panel members were recruited through digital advertisements on social media platforms (e.g., Instagram), as well as by sharing information with online parent groups on Facebook. Young people who expressed interest were first invited to a taster session, during which the aims of the planned involvement activities and the overall study were explained to help them decide whether they wanted to join the panel. The final youth panel included 14 young people aged 12–17 years old. Parental consent was obtained for all panel members and each young person received £25 per each hour of involvement (shopping voucher or bank transfer).

The initial development and refinement of the DAFI prototype was conducted in three steps. In the first step, a list of candidate items concerning digital activity and psychological reactions was drawn up by the study team based on a systematic review of the existing evidence (Kostyrka‐Allchorne et al. 2023), and consultations with experts. The proposed items aimed to capture online exposures and experiences that were relevant to mental health and wellbeing, therefore, activities that concerned schoolwork, study or paid work were not included. The candidate items list was reviewed at internal research team meeting and refined before it was shared with the youth panel.

In the second step, youth panel members were invited to list any psychological reactions they associated with digital activity using a shared online document. Their suggestions were then discussed in a group panel meeting. In the same group meeting, they also completed a card sorting task to agree which reactions should be retained and which were either irrelevant to contemporary youth online experiences or redundant and could be removed. For example, reactions such as ‘bored’, ‘free’ and ‘authentic’ were judged to be vague and hard to understand in relation to online experiences, or less relevant in relation to mental health and were removed from the prototype. After the meeting, the refined candidate list of activities and reactions was emailed to the youth panel with a request to make the final suggestions and edits to ensure that the prototype DAFI comprised items that were relevant and covered a comprehensive range of online experiences. This was a ‘homework task’ and the panel members completed it in their own time.

The new items generated by the youth panel as part of this step included both positive (e.g., *I looked for personal advice*) and negative (e.g., *I was left out of something my friends were doing online*) activities and cognitive and emotional reactions that were not identified in the review of the previous literature (e.g., *recharged, attractive, ugly, insecure*). The youth panel also highlighted activities that were *relevant* and should remain in the prototype DAFI (e.g., *chatted with family/friends, played an online game*).

In the final step, youth panel members completed the DAFI prototype during the online session and provided feedback on the questionnaire. For example, most young people did not understand the term ‘venting’ and the item was revised to ‘venting/ranting’, which was considered acceptable. All feedback from the youth panel was documented and used to finalise the DAFI prototype.

The resulting DAFI prototype included 32 items measuring digital activity and 40 items measuring feelings. This number was further reduced to 23 activity items and 24 psychological reactions items based on the preliminary review of the item‐by‐item frequency data and in consultation with the youth panel to ensure that the remaining activities were relevant to young people and captured emotions and cognitions relevant to digital experience rather than domain‐general psychological reactions.

### 2.2 Scale Psychometric Properties and Testing Predictive Validity

#### 2.2.1 Participants

Two convenience samples of older adolescents were recruited from secondary schools and two universities in England. Sample one age ranged from 16 to 20 years, and sample two from 18 to 25 years. To recruit the younger sample, information about the study was shared with three secondary schools in England within the researchers' professional networks. Each school was contacted by email with an invitation to participate in a research study on adolescent technology use and mental health. The invitation letter included details about the overall aim of the research, the institutions involved, and the funder. It explained that schools could support the research by either sharing a digital recruitment pack with potentially eligible students or allowing researchers to visit the school to promote the study or collect data. The older sample was recruited through the respective universities' Sona recruitment systems and volunteer circular emails. Ethical approval was received from London School of Economics and Political Science for the younger sample (reference 18934) and for Queen Mary University of London (reference PSY2023‐39A) for the older sample. All participants provided written consent online.

#### 2.2.2 Measures

##### 2.2.2.1 Online Exposure and Experiences

Digital activity and psychological reactions were measured with the DAFI, which has two sections: *My Online Activity* and *My Feelings Online. My Online Activity* has 23 items covering likely positive (e.g., ‘I played games with others’), neutral (e.g., ‘I liked or shared other people's posts’) and negative (e.g., ‘I saw people talk about or show ways of being very thin’) digital activities and experiences. Young people rated how often they did/experienced each in the past 2 weeks on a 5‐point scale (0 = ‘never” to 4 = ‘at least every day’). *My Feelings Online* includes 24 positive and negative items ‐ both general affective reactions (e.g., ‘stressed’, ‘calm’) and those which are more cognitive, concerning the perception of self (e.g., ‘insecure’, ‘attractive’, ‘loved’). For each item, adolescents rated how often being online made them feel this way in the past 2 weeks (0 = ‘never’ to 4 = ‘at least every day’).

Screen time was measured with a single question: ‘*On an ordinary day, about how long do you spend on your phone or the internet (not counting time for study/work)?’* (1 = ‘little or no time’ to 9 = ‘about 7 h’).

##### 2.2.2.2 Mental Health and Wellbeing

The younger sample completed the Revised Child Anxiety and Depression Scale 25–Youth Version (RCADS‐25) (Ebesutani et al. 2017), a 25‐item questionnaire that measures the severity of anxiety and depression scores. Participants rated how often each statement applied to them (0 = ‘never’ to 3 = ‘always’). Scores for the Total Anxiety (15 items, e.g., ‘I think about death’) and Total Depression subscale (10 items, e.g., ‘I have no energy for things’) were calculated. The older sample completed the Depression, Anxiety, and Stress Scale (DASS‐21) (Lovibond and Lovibond 1995), a validated 21‐item questionnaire measuring adult depression, anxiety and stress. The respective depression and anxiety subscales consist of seven items (0 = ‘did not apply to me at all’ to 3 = ‘applied to me very much, or most of the time’).

The Warwick‐Edinburgh Mental Wellbeing Scale (WEMWBS) (Tennant et al. 2007) measured wellbeing. This 14‐item positively worded questionnaire assesses subjective psychological functioning (e.g., ‘I've been feeling confident’) and all items were rated on a scale ranging from 0 = ‘none of the time’ to 5 = ‘all of the time’. A total score was calculated.

##### 2.2.2.3 Other Variables

Participants provided information about their age, sex, gender, ethnicity, and present and past eligibility for free school meals (a proxy for low socio‐economic status).

#### 2.2.3 Procedure

After informed consent was given, the data were collected online via two established web‐based survey tools: Qualtrics (https://www.qualtrics.com; the younger sample) or Gorilla (https://www.gorilla.sc; the older sample) between April 2023 and March 2024. Participants could skip questions they did not wish to answer. Participants in the younger sample completed the DAFI twice, approximately a week apart, to allow for test‐retest analysis. After completion, all participants received a £10 shopping voucher or three course credits, depending on their preference, as compensation for participating in the study.

#### 2.2.4 Analysis Strategy

First, means and standard deviations (SD) were calculated for the DAFI activities and psychological reaction items, which were then ranked from the most to the least frequent. Second, to examine the latent structure of the items, exploratory factor analysis (EFA) using the principal axis factoring method with direct oblimin rotation was performed. The generated factors were extracted based on an eigenvalue (≥ 1), the scree plot, the proportion of variance explained by each factor and their conceptual coherence to establish the optimal number of factors. When the scree plot/variance explained or conceptual coherence of items within the factor suggested that the optimal number of factors should be different to that generated based on an eigenvalue of ≥ 1, the EFA was re‐run to generate the pre‐determined number of factors. Items with loadings > 0.4 on more than one factor or items with loadings < 0.4 were removed from resulting subscales. The reliability of the subscales was assessed by calculating their internal consistency using McDonald's omega (*ϖ*) (Hayes and Coutts 2020) and the rules of thumb were applied: > 0.90 = excellent, > 0.80 = good, > 0.70 acceptable, > 0.60 = questionable, > 0.50 = poor, and < 0.50 = unacceptable. Subscale scores were calculated by averaging individual item scores. Test‐retest was estimated using Pearson's correlations.

Next, the mental health scores measured with the RCADS‐25 and the DASS‐21 were transformed into standardised z‐scores before being included in the analysis. Bivariate associations between DAFI subscales, screen time and mental health and wellbeing scores were calculated using Pearson's correlations. Rules of thumb proposed by Cohen (1988) were applied to interpret the magnitude of correlations (i.e., 0.10 < *r* < 0.30 = ‘weak’; 0.30 < *r* < 0.50 = ‘moderate’ and *r* > 0.50 = ‘strong’). Finally, the independent contribution of the DAFI subscales scores and screen time in explaining variations in depression, anxiety and wellbeing was then tested using stepwise linear multiple regressions. In step 1, the outcome was regressed on only screen time. In step 2, the DAFI digital activities were added as exposure variables; in step 3, the DAFI psychological reactions were also included as exposure variables. This stepped approach was used to explore (1) whether associations with screen time persisted once the DAFI digital activities subscales were added and (2) to establish the independent contribution of the DAFI activities and reactions as the predictors of mental health and wellbeing. Because significant mean differences were found in the digital activities and psychological reactions subscale scores between males and females, (see Supporting Information S1: Table S1), regression analyses controlled for sex by using residuals (for regression analysis results without controlling for sex, see Supporting Information S1: Table S2). There were no significant differences between the two age groups.

## 3 Results

The study included 383 participants (289 females [75.5%], 91 males [23.8%]; mean age 19 years [SD = 1.7], Table 1) from a range of ethnic communities: 187 (48.8%) of participants were White, 99 (25.8%) were South Asian, 33 (8.6%) were Black, 17 (4.4%) East Asian and 40 (10.5%) reported mixed or ’other’ ethnicity. Overall, 64 (16.8%) participants reported having received free school meals (the national average for England in 2023 was 23.8%; Department for Education 2024). Average reported daily screen time was 4.6 h (SD = 1.6).

**TABLE 1:** Participants characteristics, screen time, mental health and wellbeing scores.

Characteristic	Total (*n* = 383)	Mean (SD)	Minimum	Maximum
Age in years	378 (98.7)	19.0 (1.7)	16	25
Participant sex				
Female	289 (75.5)			
Male	91 (23.8)			
Participant ethnicity				
Black	33 (8.6)			
East Asian	17 (4.4)			
South Asian	99 (25.8)			
White	187 (48.8)			
Other' ethnicity	19 (5.0)			
Received free school meals	64 (16.8)			
Screen time	383 (100)	6.6 (1.6)	2	9
Mental health and wellbeing				
Depression symptoms (RCADS‐25)	191 (49.9)	11.80 (7.28)	0	30
Anxiety symptoms (RCADS‐25)	191 (49.9)	13.95 (9.64)	0	45
Depression symptoms (DASS‐21)	186 (48.6)	26.91 (10.00)	14	54
Anxiety symptoms (DASS‐21)	187 (48.8)	27.06 (9.34)	14	54
Wellbeing score	374 (97.7)	43.63 (9.95)	14	70

Abbreviations: DASS‐21 = the depression, anxiety, and stress scale; RCADS‐25 = the revised child anxiety and depression scale 25–youth version.

Table 2 presents the mean frequency scores for specific digital activities over the previous 2 weeks, ranked from the most to least frequent, which varied considerably for different activities. The most frequent activities were putatively positive/neutral (e.g., chatted with family/friends, did a hobby). Less frequent were activities that were more putatively negative (e.g., treated in a hurtful way, saw gory/violent images). Table 3 presents the mean frequency scores for psychological reactions to being online in the previous 2‐week period, ranked from most to least frequent. Positive reactions (e.g., loved, calm, safe, supported) were experienced more frequently than negative (e.g., hated, rejected, helpless).

**TABLE 2:** Digital activities ranked from the most to the least frequent.

Rank	Activity	Mean score (SD)
Rated more than ‘most days’		
1	Chatted with family/friends	3.37 (0.90)
2	Watched fun/positive content	3.12 (0.91)
Rated more than ‘a few times a week’		
3	Liked/shared others' posts	2.82 (1.21)
4	Checked online to see what others were doing	2.11 (1.25)
Rated more than ‘once or twice’ over 2 weeks		
5	Compared myself with how others look	1.80 (1.36)
6	Did a hobby	1.78 (1.25)
7	Saw someone venting/ranting	1.77 (1.26)
8	Did something fun online with family/friends	1.62 (1.26)
9	Compared myself with how others socially	1.57 (1.38)
10	Watched some dark/negative content	1.48 (1.22)
11	Saw people being hateful to groups/individuals	1.26 (1.21)
12	Looked for personal advice	1.23 (1.22)
13	Played an online game with other people	1.19 (1.35)
14	Saw people talk about ways of being very thin	1.09 (1.24)
15	Saw sexual images/video	0.95 (1.06)
Rated less than ‘once or twice’ over 2 weeks		
16	Saw gory/violent image or video	0.93 (1.26)
17	Used an app/website to improve mental health	0.93 (1.09)
18	Saw people talk about/show illegal drugs	0.86 (1.08)
19	Chatted online with someone I don't really know	0.75 (1.04)
20	Was left out of things my friends were doing online	0.59 (0.99)
21	Saw ways of physically hurting themselves	0.44 (0.84)
22	Was treated in a hurtful or nasty way	0.33 (0.73)
23	Met in person with someone I got to know online	0.33 (0.81)

**TABLE 3:** Psychological reactions ranked from the most to the least common.

Rank	Reaction	Mean score (SD)
Rated more than ‘a few times a week’		
1	Loved	2.04 (1.28)
2	Calm	2.00 (1.08)
3	Safe	1.99 (1.31)
4	Supported	1.96 (1.20)
5	Included	1.94 (1.19)
Rated more than ‘once or twice’ over 2 weeks		
6	Drained	1.88 (1.32)
7	Understood	1.85 (1.14)
8	Overwhelmed	1.79 (1.31)
9	Hopeful	1.79 (1.10)
10	Stressed	1.77 (1.28)
11	Confident	1.69 (1.12)
12	Insecure	1.67 (1.36)
13	Valued	1.66 (1.16)
14	Worried	1.58 (1.25)
15	Free	1.58 (1.22)
16	Lonely	1.55 (1.31)
17	Recharged	1.47 (1.18)
18	Upset	1.42 (1.15)
19	Attractive	1.41 (1.13)
20	Ugly	1.37 (1.32)
21	Judged	1.27 (1.20)
Rated as less than ‘once or twice’		
22	Helpless	1.08 (1.231)
23	Rejected	0.83 (1.103)
24	Hated	0.76 (1.114)

Exploratory factor analysis of the digital activity items suggested that 20 items should be retained within five factors. Three were putatively negative and potentially harmful: *Risky Content* (7 items) ‐ seeing negative events/images online; *Social Comparison* (3 items) ‐ comparing one's appearance or popularity to others; and *Risky Interactions* (4 items) ‐ being the subject of negative treatment online or interacting with strangers in potentially inappropriate ways. Two factors were more positive: *Leisure Activities* (3 items) ‐ online hobbies or games; and *Social Engagement* (3 items) ‐ interactions with others (Table 4).

**TABLE 4:** Item‐to‐factor loading of digital activity items.

Items	Risky content	Leisure activities	Social engagement	Social comparison	Risky interactions
Watched some dark/negative content	**−0.682**	−0.058	0.039	−0.072	−0.051
Saw gory/violent image or video	**−0.676**	0.043	−0.030	0.040	0.061
Saw people being hateful to certain groups/individuals	**−0.592**	0.035	0.026	0.069	0.030
Saw someone venting/ranting	**−0.456**	−0.202	0.270	−0.087	0.138
Saw people talk about or show illegal drugs	**−0.455**	0.002	0.117	−0.051	0.211
Saw a sexual image or video	**−0.446**	0.186	−0.016	−0.156	0.041
Saw people talk about physically hurting themselves	**−0.441**	0.115	−0.138	−0.138	0.286
Did something fun online with family or friends	0.070	**0.669**	0.236	−0.001	0.080
Played an online game with other people	−0.014	**0.624**	−0.125	0.017	0.045
Did a hobby	−0.065	**0.474**	0.026	0.035	−0.060
Watched some fun/positive content	−0.010	0.048	**0.634**	0.103	−0.055
Chatted with family/friends	0.053	0.018	**0.539**	−0.036	0.044
Liked or shared other people's posts	−0.207	0.000	**0.484**	−0.039	−0.081
Compared myself with how others look	0.020	−0.094	−0.049	**−0.918**	−0.052
Compared myself with how others are doing socially	−0.073	0.003	−0.086	**−0.858**	−0.029
Saw people talk about or show ways of being very thin	−0.048	0.002	0.037	**−0.503**	0.168
Was treated in a hurtful or nasty way	0.001	−0.096	−0.008	0.078	**0.844**
Met in person with someone I got to know online	−0.011	0.077	−0.037	−0.032	**0.549**
Was left out of something my friends were doing	−0.081	−0.019	−0.050	−0.068	**0.514**
Chatted online with someone I don't really know	−0.113	0.141	0.025	−0.043	**0.436**
Checked online to see what other people were doing	−0.131	−0.029	0.356	−0.288	−0.049
Looked for personal advice	−0.074	−0.068	0.213	−0.306	0.184
Used an app or website to improve my mental health	0.128	0.123	0.198	−0.259	0.136

*Note:* Factor loadings above 0.4 which did not cross‐load are bolded.

Exploratory factor analysis of the psychological reaction items suggested that all 24 items should be retained within three factors. *Negative Self‐Reactions* (7 items) ‐ negative feelings and thoughts about oneself after being online (e.g., ‘lonely’, ‘insecure’, ‘hated’); *Positive Reactions* (12 items) ‐ positive emotional states and thoughts and feelings about oneself; and *Negative Stress Reactions* (5 items) ‐ general negative reactions, feeling ‘overwhelmed’ and ‘worried’ (Table 5).

**TABLE 5:** Item‐to‐factor loadings of psychological reaction items.

Item	Negative self‐reactions	Positive reactions	Negative stress reactions
Rejected	**0.773**	0.070	0.061
Hated	**0.731**	0.012	−0.008
Judged	**0.704**	0.080	−0.058
Lonely	**0.600**	−0.067	−0.137
Ugly	**0.596**	−0.130	−0.131
Insecure	**0.520**	−0.079	−0.219
Helpless	**0.417**	−0.035	−0.347
Valued	0.042	**0.777**	−0.085
Confident	−0.013	**0.758**	0.035
Supported	−0.030	**0.736**	−0.069
Loved	−0.170	**0.716**	−0.219
Hopeful	0.061	**0.643**	−0.143
Safe	0.083	**0.630**	0.059
Understood	−0.041	**0.630**	−0.135
Included	−0.037	**0.607**	−0.050
Attractive	−0.122	**0.606**	−0.035
Calm	−0.022	**0.603**	0.179
Free	0.069	**0.585**	0.151
Recharged	0.115	**0.564**	0.179
Overwhelmed	0.037	0.051	**−0.758**
Worried	0.116	0.023	**−0.682**
Stressed	0.171	0.062	**−0.665**
Drained	0.246	0.009	**−0.562**
Upset	0.290	−0.073	**−0.560**

*Note:* Factor loadings above 0.4 which did not cross‐load are bolded.

The internal consistency (McDonald's *ϖ*) of the digital activity subscales ranged from > 0.70 for *Risky Content, Risky Interactions* and *Social Comparison* to 0.62 and 0.57 for *Leisure Activities* and *Social Engagement*, respectively. The internal consistency (McDonald's *ϖ*) of the three reaction subscales were all > 0.85. For digital activities, test‐retest reliability was high at the subscale level: *Risky Content*, *r* = 0.85; *Leisure Activity*, *r* = 0.75; *Social Engagement*, *r* = 0.68; *Social Comparison*, *r* = 0.75; *Risky Interactions*, *r* = 0.76. For psychological reactions, test‐retest reliability was also high: *Negative Self‐Reactions—r* = 0.79; *Positive Reactions—r* = 0.61; *Negative Stress Reactions—r* = 0.78.

Descriptive information about screen time, digital activity and psychological reactions, depression and anxiety symptoms and wellbeing is reported in Table 6.

**TABLE 6:** Mean subscale scores (calculated by averaging individual item scores) for digital activity and psychological reactions measured with the DAFI.

DAFI subscale	Total (*n* = 383)	Mean (SD)	Minimum	Maximum
Digital activities				
Risky content	383 (100)	1.1 (0.8)	0	3.9
Leisure activities	383 (100)	1.5 (1.0)	0	4.0
Social engagement	383 (100)	3.1 (0.7)	0	4.0
Social comparison	383 (100)	1.5 (1.1)	0	4.0
Risky interactions	383 (100)	0.5 (0.7)	0	3.8
Reactions				
Negative self‐reactions	383 (100)	1.2 (0.9)	0	4.0
Positive reactions	383 (100)	1.8 (0.8)	0	4.0
Negative stress reactions	382 (99.7)	1.7 (1.0)	0	4.0

Abbreviation: DAFI = the digital activity and feelings inventory.

Table 7 presents the bivariate correlations between subscale scores for digital activity and psychological reactions to being online. *Negative Self Reactions* subscale was strongly and positively correlated with all three putatively negative activities subscales, namely, *Risky Content, Social Comparison* and *Risky Interactions* (*r* = 0.53, *r* = 0.63 and *r* = 0.48, respectively). It was also positively correlated with screen time albeit weakly (*r* = 0.15). *Positive Reactions* subscale was moderately and positively correlated with *Leisure Activities* and *Positive Interactions* (*r* = 31 and *r* = 22, respectively). *Negative Stress Reactions* were most strongly correlated with *Risky Content* and *Social Comparison* (*r* = 0.48 and *r* = 0.45, respectively) and to a lesser extent with *Risky Interactions* and *Social Engagement* (*r* = 0.29 and *r* = 0.20, respectively).

**TABLE 7:** Bivariate correlations between digital activities, psychological reactions and screen time.

	Negative	Positive	Negative stress reactions
	self‐reactions	reactions	
Risky content	0.52**	0.03	0.48**
Leisure activities	−0.09	0.31**	−0.07
Social engagement	0.10*	0.22**	0.20**
Social comparisons	0.63**	−0.06	0.45**
Risky interactions	0.48**	0.11*	0.29**
Screen time	0.15**	0.04	0.20**

^*^ Correlation significant at uncorrected alpha (*p* < 0.05).

^**^ Correlation significant at corrected alpha (*p* < 0.016).

Further, depression and anxiety symptoms were significantly positively correlated with screen time but with weak magnitude for both variables (*r* < 0.20). Depression and anxiety symptoms were both positively moderately correlated with *Risky Content, Risky Interactions* and *Social Comparison* (rs > 0.35, Table 8). Both were also strongly correlated with *Negative Self Reactions* and *Negative Stress Reactions* (rs > 0.50, Table 8). Depression but not anxiety symptoms were weakly negatively correlated with *Leisure Activities* (*r* = −0.10) and *Positive Reactions* (*r* = −0.17). Wellbeing was weakly negatively correlated with screen time, *Risky Content, Risky Interactions* and *Social Comparison (rs < −0.25). It was also moderately negatively correlated with Negative Self‐Reactions* and *Negative Stress Reactions (r = −0.40 and r = −0.29, respectively)* and positively correlated with *Leisure Activities and Positive Reactions (r = 0.19 and r = 0.41, respectively)*.

**TABLE 8:** Correlations between digital activities, psychological reactions and screen time and depression and anxiety symptoms, and wellbeing.

	Depression	Anxiety	Wellbeing
Digital activities			
Risky content	0.44**	0.39**	−0.17**
Leisure activities	−0.10*	−0.07	0.19**
Social engagement	0.07	0.09	0.09
Social comparison	0.51**	0.50**	−0.24**
Risky interactions	0.36**	0.36**	−0.11*
Reactions			
Negative self‐reactions	0.65**	0.60**	−0.40**
Positive reactions	−0.17**	−0.05	0.41**
Negative stress reactions	0.53**	0.51**	−0.29**
Screen time	0.18**	0.11*	−0.16**

^*^ Correlation significant at uncorrected alpha (*p* < 0.05).

^**^ Correlation significant at corrected alpha (*p* < 0.016).

Finally, in linear regression analyses, in step 1, screen time was positively associated with depression symptoms (*β* = 0.16). In step 2, when the DAFI activities subscales were added to the model, only *Social Comparison* (*β* = 0.32), *Risky Content* (*β* = 0.29), *Social Engagement* (*β* = −0.14), and *Risky Interactions* (*β* = 0.12) were significantly associated with depression symptoms. When the DAFI reactions were added in step 3, *Risky Content* (*β* = 0.15), *Social Comparison* (*β* = 0.10) and *Social Engagement* (*β* = −0.09) remained significantly associated with depression symptoms but with much reduced magnitude. *Negative Self Reactions* (*β* = 0.39) and, with lesser magnitude, *Positive Reactions* (*β* = −0.11) were associated with depression symptoms (Table 9). The results for anxiety symptoms mirrored those for depression. The strongest positive correlates in step 2 were *Social Comparison* and *Risky Content* (*β* = 0.30 and *β* = 24, respectively), followed by *Risky Interactions* and low *Social Engagement* (*β* = 0.14 and *β* = −0.10, respectively). The magnitude of all associations reduced substantially in step 3 when the reactions subscales were added. *Negative Self Reactions* once again was the strongest correlate in the final model (*β* = 0.30). *Negative Stress Reactions* was also a weak significant correlate (*β* = 0.15). For both outcomes, the association with screen time was negligible (Table 9).

**TABLE 9:** Regression analyses of associations between screen time, digital activities and psychological reactions and depression and anxiety symptoms and wellbeing controlling for sex.

	Depression						Anxiety						Wellbeing					
	Step 1		Step 2		Step 3		Step 1		Step 2		Step 3		Step 1		Step 2		Step 3	
	*β*	*p*	*β*	*p*	*β*	*p*	*β*	*p*	*β*	*p*	*β*	*p*	*β*	*p*	*β*	*p*	*β*	*p*
Screen time	**0.16**	**0.002**	0.07	0.15	0.05	0.278	0.09	0.08	0	0.98	−0.02	0.622	**−0.15**	**0.003**	**−0.15**	**0.004**	**−0.13**	**0.004**
Risky content			**0.29**	**<** **0.001**	**0.15**	**0.005**			**0.24**	**<** **0.001**	0.11	0.061			−0.12	0.062	0.01	0.825
Leisure activities			−0.08	0.082	0.03	0.469			−0.04	0.439	0.03	0.495			**0.16**	**0.003**	0.00	0.965
Social engagement			**−0.14**	**0.002**	**−0.09**	**0.039**			**−0.10**	**0.038**	−0.08	0.086			**0.19**	**<** **0.001**	**0.10**	**0.046**
Social comparison			**0.32**	**<** **0.001**	**0.10**	**0.049**			**0.30**	**<** **0.001**	**0.13**	**0.021**			**−0.18**	**0.001**	0.02	0.741
Risky interactions			**0.12**	**0.023**	0.06	0.253			**0.14**	**0.008**	0.08	0.118			−0.02	0.758	0.02	0.767
Negative self‐reactions					**0.39**	**<** **0.001**					**0.30**	**<** **0.001**					**−0.34**	**<** **0.001**
Positive reactions					**−0.11**	**0.009**					0.00	0.924					**0.35**	**<** **0.001**
Negative stress‐reactions					0.11	0.064					**0.15**	**0.019**					−0.05	0.488
F(df) for Δ*R* ^2^	(1, 371) 9.82**		(5, 366) 35.01**		(3363) 26.19**		(1, 372) 3.05		(5, 367) 27.48**		(3.364) 16.52**		(1, 368) 8.68**		(5, 363) 9.20**		(3, 360) 29.35**	
Δ*R* ^2^	0.03		0.32		0.12		0.01		0.27		0.09		0.02		0.11		0.17	
Adjusted *R* ^2^	0.02		0.33		0.45		0.01		0.27		0.35		0.02		0.12		0.29	

*Note:* Significant contributors to each model at each step to 0.05 are bolded.

*p < 0.05.

** *p* < 0.01.

Wellbeing showed a different pattern of results. First, screen time was a significant predictor of wellbeing at all three steps, with the small effect size remaining unchanged by adding DAFI subscale scores to the model (*β* = −0.15 to *β* = −0.13). Second, in step 2, *Social Engagement* and *Leisure Activity* were both significant weak positive correlates (*βs* < 0.20) *and Social Comparison* was a significant negative predictor (*β* = −0.18). Once DAFI reactions subscale scores were added in step 3, only *Social Engagement* remained significantly associated with wellbeing (*β* = 0.10). In this step, the strongest associations were with *Negative Self Reactions* (*β* = −0.34) and *Positive Reactions* (*β* = 0.35).

## 4 Discussion

The public and academic debate about the impact of digital technology on adolescent mental health and wellbeing has often been informed by the results of studies using reductive measures of screen time. Such measures are limited because they (i) do not differentiate between the effects of different activities and (ii) do not consider the affective and cognitive impact of being online, which clinical models highlight as key mediators of poor mental health (i.e., depression and anxiety). In the present study, we developed and examined the psychometric properties of a new self‐report measure, the DAFI, specifically developed to estimate both engagement in specific digital activities and affective and cognitive reactions to being online. We also tested the DAFI's validity to predict variations in symptoms of depression, anxiety and general psychological wellbeing relative to a typical measure of screen time. The analyses revealed six important findings.

First, participants reported engaging in positive digital activities more often than negative activities. They also reported that their digital activity made them feel positive more often than negative. The data are not, therefore, consistent with the public perception that being online is dominated by negative activities and experiences for adolescents or that risks are pervasive and widely encountered. Here, some caution is advised, given that self‐reported digital activity may be liable to social desirability and recall biases (Browne et al. 2021). Nevertheless, this finding aligns with proposals that digital technology, more broadly, could be an important source of social and psychological support for youth (Odgers and Jensen 2020).

Second, exploratory factor analysis produced a psychometrically sound measure of online experiences consisting of 44 items: 20 digital activity items which loaded on five factors and 24 psychological reactions that loaded on three factors. Although the DAFI was designed to be used in studies with adolescent respondents, results from the present study that recruited youth sample suggest that the measure can also be an effective tool to measure digital activities and psychological reactions to being online in young adults (though, 64% were 19 years old or younger and only 3 participants were older than 24 years).

Importantly, the DAFI proved an effective tool for distinguishing potentially beneficial or risky digital activities, as well as positive and negative reactions to being online. It also provided a granular characterisation of both activities and emotional reactions. Within the risky digital activities, the DAFI distinguished exposure to risky content (e.g., seeing potentially upsetting events or encountering harmful content online) from making unfavourable social comparisons or engaging in risky interactions (e.g., being treated in a hurtful or nasty way). In terms of psychological reactions to being online, the DAFI distinguished negative self‐reactions (i.e., how one feels about themselves, e.g., ‘rejected’, ‘lonely’, ‘hated’) from negative stress‐reactions (i.e., more general negative emotions, e.g., ‘stressed’, ‘overwhelmed’). Within the beneficial activities, the DAFI distinguished between digital activities that have a more social character (e.g., ’chatted with family/friends’, ’liked or shared other people's posts’) and those that describe online leisure (e.g., hobbies or games). The face validity of the distinctive risk clusters identified using the DAFI is supported by prior literature highlighting the mental health risks associated with risky content (Kostyrka‐Allchorne et al. 2023), risky interactions (Boer et al. 2021) and social comparison (Marciano et al. 2024; Orben et al. 2024).

Third, despite the young people reporting less frequent negative online experiences and reactions, these were more strongly associated with increased symptoms of anxiety and depression. In particular, the more frequent *Social Comparison* and exposure to *Risky Content* were strongly associated with poorer mental health. In contrast, more frequently experienced positive online activities, such as *Social Engagement,* were only weakly associated with reduced symptoms of depression and anxiety, while *Leisure Activities* were not associated with mental health outcomes. Crucially, the finding that specific digital activities subscales were differentially associated with mental health outcomes highlights the value of distinguishing specific digital activities to isolate risk from non‐risk factors.

Fourth, while the digital correlates of anxiety and depression symptoms were similar, they were different from those of wellbeing. That is, wellbeing could not simply be considered as the obverse of poor mental health in relation to digital activity and psychological reactions to being online. Positive activities, especially *Social Engagement*, significantly predicted wellbeing, more strongly than the absence of digital risk did.

Fifth, emotional reactions to being online were more powerful as predictors than self‐reported digital activity across all outcomes. Crucially, *Negative Self‐Reactions* were strongly correlated with increased symptoms of depression and anxiety and reduced wellbeing. *Positive Reactions* were associated with improved wellbeing. *Negative Stress Reactions* played little or no role as a meaningful correlate of the examined outcomes. In this sense, the findings are more consistent with cognitive rather than stress‐driven models of the role of social experience on mental health risks (Beck 2002; Kessler 1997). Interestingly, once these reactions were included in the regression models, the potential predictive power of the digital activities subscales reduced substantially, providing initial cross‐sectional support for the hypothesis that subjective reactions mediate the impact of digital activity on mental health (Sonuga‐Barke et al. 2024).

Finally, the results support our hypothesis that the DAFI provides greater predictive power to detect variations in mental health than screen time. In this study, associations between screen time and mental health were negligible and not significant once DAFI subscales were added to the regression models. Based on this analysis, the assessment of digital risks provides a better and more granular assessment of how adolescents' digital activities contribute to mental ill‐health. Especially noteworthy are the exposure to *Risky Content*, the engagement in *Social Comparison*, and the negative cognitions and emotions that being online evokes, particularly in relation to the negative evaluation of the self. Screen time appears to play a greater role in undermining wellbeing, though the effects observed were still small.

### 4.1 Limitations

Although the current report highlights the value of the DAFI, the study had several limitations. First, the current analysis was based on cross‐sectional data, which limits the causal inference based on the direction of effects. It is quite possible that digital activity and psychological reactions are shaped by pre‐existing levels of depression and anxiety rather than causing them. Recently, we have highlighted a transactional model of the complex and reciprocal relations between digital activity and mental health in which digital activity drives anxiety and depression and, in turn, increased anxiety and depression levels lead to more online risk exposure and more negative reactions to being online (Sonuga‐Barke et al. 2024). We are currently undertaking a longitudinal study to disentangle these factors (Kostyrka‐Allchorne et al. 2024). Second, digital activity, mental health and wellbeing estimates were based on self‐report measures, with no independent or objective validation of digital activity self‐report. This leaves the possibility of shared method variance artificially elevating the size of the associations between the variables examined in this study. However, given the differentiated profile of correlations, it appears unlikely that shared method variance could account for the current pattern of results. Third, we employed a convenience sample which did not distinguish levels of clinical depression or anxiety. Future studies could seek to replicate the findings in a group with more severe mental health difficulties. Finally, females were over‐represented in this convenience sample as were certain ethnic groups with respect to the age matched UK population.

## 5 Conclusion

Measuring digital activity and associated reactions using the DAFI increased our ability to detect variations in mental health and wellbeing, over and above screen time, highlighting the potential role of risky content, social comparison and associated negative self‐reactions as influential factors in adolescent mental ill‐health.

## Author Contributions

**Katarzyna Kostyrka‐Allchorne:** conceptualization, writing – original draft, writing – review and editing, funding acquisition, supervision. **Jake Bourgaize:** conceptualization, writing – review and editing, data curation, formal analysis, project administration. **Aja Murray:** conceptualization, writing – review and editing. **Mariya Stoilova:** conceptualization, writing – review and editing, project administration. **Iqra Abbas:** writing – review and editing, data curation, project administration. **Amy Bridgwood:** conceptualization, writing – review and editing, data curation, project administration. **Eliz Azeri:** conceptualization, writing – review and editing, data curation. **Chris Hollis:** writing – review and editing, funding acquisition. **Ellen Townsend:** writing – review and editing, funding acquisition. **Sonia Livingstone:** conceptualization, writing – original draft, writing – review and editing, funding acquisition, supervision. **Edmund J. S. Sonuga‐Barke:** conceptualization, writing – original draft, writing – review and editing, funding acquisition, supervision. [Correction added on 17th July 2025, after first online publication: In the Author Contributions, the first names and surnames of the first 10 authors have been corrected to match the corrected author name formatting.]

## Ethics Statement

Ethical approval was received from London School of Economics and Political Science for the younger sample (reference 18934) and Queen Mary University of London (reference PSY2023‐39A) for the older sample.

## Consent

All participants provided written consent online.

## Conflicts of Interest

The authors declare no conflicts of interest.

## Permission to Reproduce Material From Other Sources

The authors have nothing to report.

## Supporting information

Supporting Information S1

## Data Availability

The data will be deposited in a public archive within 2 years after the end of the project (current project end date: 31 August 2025).
