# Correction to “Understanding Youth Online Experiences and Mental Health: Development and Validation of the Digital Activity and Feelings Inventory (DAFI)”

**DOI:** 10.1002/mpr.70030

**Published:** 2025-07-17

Kostyrka‐Allchorne, K., J. Bourgaize, A. Murray, et al. 2025. “Understanding Youth Online Experiences and Mental Health: Development and Validation of the Digital Activity and Feelings Inventory (DAFI).” *International Journal of Methods in Psychiatric Research* 34, no. 2: e70028. https://doi.org/10.1002/mpr.70028.

In the above article, all the authors' names, except the last author and the study group, were incorrectly listed, with the authors' first names displaying as surnames. The correct citation should read:

Kostyrka‐Allchorne, K., Bourgaize, J., Murray, A., Stoilova, M., Abbas, I., Bridgwood, A., Azeri, E., Hollis, C., Townsend, E., Livingstone, S., Sonuga‐Barke, E.J.S., on behalf of the Digital Youth Research Programme 2025. “Understanding Youth Online Experiences and Mental Health: Development and Validation of the Digital Activity and Feelings Inventory (DAFI).” *International Journal of Methods in Psychiatric Research* 34, no. 2: e70028. https://doi.org/10.1002/mpr.70028.

Additionally, the author names have also been corrected in the funding statement and the author contributions.

The online version of this article has been corrected accordingly.

We apologize for this error.

