# Supplementary material for: Understanding Youth Online Experiences and Mental Health: Development and Validation of the Digital Activity and Feelings Inventory (DAFI)
Source: Int J Methods Psychiatr Res. 2025 Jun 17;34(2):e70028. doi: 10.1002/mpr.70028 (PMC12173090; doi:10.1002/mpr.70028)
Supplement: Supplementary file 1 — Supporting Information S1 [file MPR-34-e70028-s001.docx]

**Supplementary materials**

**Sex differences in digital activity and psychological reactions.**

Mean Digital Activity and Feelings Inventory (DAFI) subscale scores and screen time (in hours) for females and males are presented in Table S1. Independent samples t-test showed that females scored significantly higher for both *Social Engagement* and *Social Comparison*. Males scored significantly higher for *Leisure activities*. In terms of psychological reactions, females scored significantly higher on both *Negative Self-Reactions* and *Negative Stress Reactions* and males scored significantly higher on *Positive Reactions*. There were no significant differences between males and females in the amount of screen time.

***Table S1.*** Mean (SD) screen time and DAFI subscale scores for males and females.

|  | **Males (n = 91)** | **Females (n = 289)** | **Direction of difference** |
| --- | --- | --- | --- |
| **Variable** | **Mean (SD)** | |  |
| Screen time (in hours) | 4.4 (1.7) | 4.7 (1.5) | ns |
| Digital activities |  |  |  |
| Risky Content | 1.1 (0.9) | 1.1 (0.7) | ns |
| Leisure Activities | **2.1 (1.1)** | **1.4 (0.9)** | **m > f**** |
| Social Engagement | **2.9 (0.8)** | **3.2 (0.7)** | **f > m**** |
| Social Comparison | **1.0 (1.0)** | **1.7 (1.11)** | **f > m**** |
| Risky Interactions | 0.5 (0.6) | 0.5 (0.7) | ns |
| Reactions |  |  |  |
| Negative Self-Reactions | **0.9 (0.9)** | **1.3 (0.9)** | **f > m**** |
| Positive Reactions | **1.9 (0.9)** | **1.7 (0.8)** | **m > f*** |
| Negative Stress Reactions | **1.3 (1.0)** | **1.8 (1.0)** | **f > m**** |

*Note: Significant differences between males and female are bolded. Asterisks denote: * p<.05; ** p<.001*

***Table S2.*** Regression analyses for associations between screen time, digital activities and reactions and depression, anxiety symptoms and wellbeing not controlling for sex.

|  | **Depression** | | | | | | **Anxiety** | | | | | | **Wellbeing** | | | | | |
| --- | --- | --- | --- | --- | --- | --- | --- | --- | --- | --- | --- | --- | --- | --- | --- | --- | --- | --- |
|  | *Step 1* | | *Step 2* | | *Step 3* | | *Step 1* | | *Step 2* | | *Step 3* | | *Step 1* | | *Step 2* | | *Step 3* | |
|  | *β* | *p* | *β* | *p* | *β* | *p* | *β* | *p* | *β* | *p* | *β* | *p* | *β* | *p* | *β* | *p* | *β* | *p* |
| Screen time | **0.18** | **<.01** | 0.09 | 0.05 | 0.07 | 0.1 | **0.11** | **0.04** | 0.01 | 0.75 | -0.01 | 0.88 | **-0.16** | **<.01** | **-0.16** | **<.01** | **-0.15** | **<.01** |
| Risky Content |  |  | **0.24** | **<.01** | 0.09 | 0.07 |  |  | **0.18** | **<.01** | 0.04 | 0.46 |  |  | -0.1 | 0.12 | 0.04 | 0.53 |
| Leisure Activities |  |  | **-0.15** | **<.01** | -0.04 | 0.37 |  |  | **-0.12** | **.<.01** | -0.05 | 0.28 |  |  | **0.19** | **<.01** | 0.03 | 0.49 |
| Social Engagement |  |  | -0.08 | 0.07 | -0.03 | 0.45 |  |  | -0.03 | 0.57 | -0.01 | 0.81 |  |  | **0.17** | **<.01** | 0.07 | 0.14 |
| Social Comparison |  |  | **0.37** | **<.01** | **0.15** | **<.01** |  |  | **0.36** | **<.01** | **0.18** | **<.01** |  |  | **-0.21** | **<.01** | 0 | 0.96 |
| Risky Interactions |  |  | 0.12 | 0.02 | 0.06 | 0.2 |  |  | **0.15** | **<.01** | 0.09 | 0.08 |  |  | -0.02 | 0.74 | 0.01 | 0.81 |
| Negative Self-Reactions |  |  |  |  | **0.37** | **<.01** |  |  |  |  | **0.29** | **<.01** |  |  |  |  | **-0.33** | **<.01** |
| Positive Reactions |  |  |  |  | **-0.12** | **<.01** |  |  |  |  | -0.01 | 0.89 |  |  |  |  | **0.35** | **<.01** |
| Negative Stress-Reactions |  |  |  |  | **0.13** | **0.02** |  |  |  |  | **0.18** | **<.01** |  |  |  |  | -0.06 | 0.39 |
| F(df) for ΔR2 | (1, 374) 12.18** | | (5, 369) 37.84** | | (3,366) 28.01** | | (1,375) 4.42* | | (5,370) 31.67** | | (3,367) 19.3** | | (1,371) 10.128** | | (5,366) 10.32** | | (3,363) 31.27** | |
| ΔR2 | 0.03 | | 0.33 | | 0.12 | | 0.01 | | 0.3 | | 0.09 | | 0.03 | | 0.12 | | 0.18 | |
| Adjusted R2 | 0.03 | | 0.35 | | 0.47 | | 0.01 | | 0.3 | | 0.39 | | 0.02 | | 0.13 | | 0.31 | |

*Note: Significant contributors to each model at each step to .05 are bolded. Asterisks denote: * p<.05; ** p<.01*
